# Surgical fasciectomy of the trapezius muscle combined with neurolysis of the Spinal accessory nerve; results and long-term follow-up in 30 consecutive cases of refractory chronic whiplash syndrome

**DOI:** 10.1186/1749-7221-5-7

**Published:** 2010-04-07

**Authors:** N Ake Nystrom, Lloyd P Champagne, Michael Freeman, Elisabet Blix

**Affiliations:** 1 Department of Orthopaedic Surgery and Rehabilitation, University of Nebraska Medical Center, Omaha, NE, USA; 2 Division of Plastic and Reconstructive Surgery, University of Nebraska Medical Center, Omaha, NE, USA; 3 Arizona Center for Hand Surgery, Phoenix, AZ, USA; 4 Department of Public Health and Preventive Medicine, Oregon Health & Science University School of Medicine, Portland, OR, USA; 5 Department of Anesthesiology, University of Pittsburgh, Pittsburgh, PA, USA

## Abstract

**Background:**

Chronic problems from whiplash trauma generally include headache, pain and neck stiffness that may prove refractory to conservative treatment modalities. As has previously been reported, such afflicted patients may experience significant temporary relief with injections of local anesthetic to painful trigger points in muscles of the shoulder and neck, or lasting symptomatic improvement through surgical excision of myofascial trigger points. In a subset of patients who present with chronic whiplash syndrome, the clinical findings suggest an affliction of the spinal accessory nerve (CN XI, SAN) by entrapment under the fascia of the trapezius muscle. The present study was undertaken to assess the effectiveness of SAN neurolysis in chronic whiplash syndrome.

**Methods:**

A standardized questionnaire and a linear visual-analogue scale graded 0-10 was used to assess disability related to five symptoms (pain, headache, insomnia, weakness, and stiffness) before, and one year after surgery in a series of thirty consecutive patients.

**Results:**

The preoperative duration of symptoms ranged from seven months to 13 years. The following changes in disability scores were documented one year after surgery: Overall pain decreased from 9.5 +/- 0.9 to 3.2 +/- 2.6 (p < 0.001); headaches from 8.2 +/- 2.9 to 2.3 +/- 2.8 (p < 0.001); insomnia from 7.5 +/- 2.4 to 3.8 +/- 2.8 (p < 0.001); weakness from 7.6 +/- 2.6 to 3.6 +/- 2.8 (p < 0.001); and stiffness from 7.0 +/- 3.2 to 2.6 +/- 2.7 (p < 0.001).

**Conclusions:**

Entrapment of the spinal accessory nerve and/or chronic compartment syndrome of the trapezius muscle may cause chronic debilitating pain after whiplash trauma, without radiological or electrodiagnostic evidence of injury. In such cases, surgical treatment may provide lasting relief.

## Background

Among patients who develop permanent debilitating symptoms after whiplash trauma (referred to as chronic whiplash syndrome henceforth), headaches and/or pain and stiffness in the neck and shoulder are the most frequent complaints and reasons for disability [1-3]. In addition, complex patterns of diffuse symptoms, including numbness, paresthesias, vertigo, muscle weakness, or cognitive dysfunction, are common and have been shown to correlate with post traumatic sleep deprivation [4] or brain stem dysfunction [5-7]. Yet, many patients claim disability in spite of normal findings on standard laboratory tests. This has led to controversy in the literature as some authors argue that symptoms are credible only if corroborated by laboratory findings [8] while others claim that negative studies do not exclude injury or the validity of a complaint [9,10].

Chronic symptoms from whiplash trauma have commonly been linked to pathology of the spine and its supporting tissues, i.e. facet joints [11], spinal ligaments [12], and intervertebral discs [13,14]. However, previous investigations have also demonstrated significant symptomatic improvement, including temporarily decreased pain, increased cervical range of motion, and higher peripheral pressure pain thresholds in chronic whiplash patients following injections of local anesthetic into carefully selected areas of focal tenderness in painful muscles [15]. The careful selection of ("key") tender points for injection appears to be critical, as previously described efforts directed at non-specific trigger points have been less effective [16].

Based upon these observations, a therapeutic approach to chronic whiplash has been developed in which offending tender points that have been identified by a positive response to infiltration with anesthetic are surgically exposed and then excised [17]. Typically, any removed tissue consisted of trapezius fascia, and thus the procedure is reasonably described as a modified fasciectomy. A central feature of the surgical strategy is that following incision and elevation of skin flaps, the patient is awakened for key portions of the procedure to provide real time feedback to assist in identifying and excising of pain generating tissue.

In a similar vein, Hagert et al. have reported that they treated chronic compartment syndrome of the trapezius and entrapment of the spinal accessory nerve (SAN) in patients with a history of overuse syndrome [18] and a clinical presentation that closely matches the pattern of symptoms observed among patients with chronic whiplash. We therefore posited peripheral nerve entrapment as a possible subcomponent of the chronic whiplash syndrome, and, in a selected group of patients undergoing the procedure described above for chronic whiplash, included neurolysis of SAN. The present manuscript describes the procedures, findings, and long-term outcome in a series of patients undergoing spinal accessory nerve decompression in combination with excision of tender points for chronic pain following whiplash.

## Methods

The study group consisted of 30 consecutive patients treated by one surgeon (NAN). The indication for surgery was established based upon the following:

• unremitting posttraumatic neck pain with a steady state for no less than six months, most typically as a result of a motor vehicle crash-related injury;

• lack of lasting response to conservative or minimally-invasive therapeutic procedures, including physical therapy, chiropractic treatment, zygapophyseal blocks, *inter alia*;

• lack of a clearly delineated pain generator pertaining to the spine, such as a disk herniation, fracture, or foraminal or central spinal stenosis;

• prompt response to anesthetic infiltration of key tender points in the region of the upper trapezius muscle (at least 50% reduction of the most intrusive symptoms).

### Anatomical considerations

**Figure 1:**
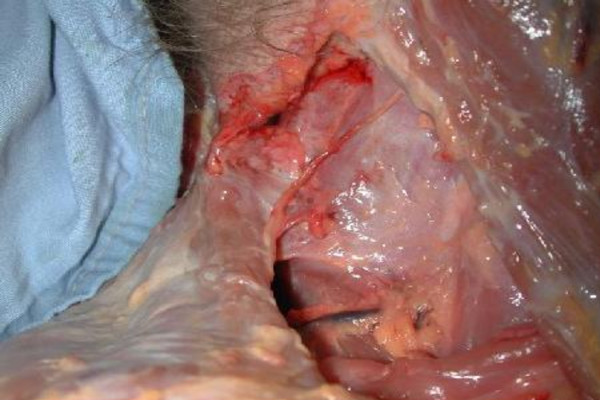
**Trajectory of the spinal accessory nerve in the posterior triangle (cadaveric dissection)**.

During its extracranial course, the SAN forms a plexus with fibers from spinal nerves C3 and C4 [19] before traversing the posterior triangle. In order to minimize the risk of surgical complications during exploration of the ventral aspect of the trapezius, the nerve must be exposed and protected (Figure 1).

### Surgical technique

Patients are placed in a lateral or beach-chair position on the operating table. Under local anesthesia (1-3 cc of Lidocaine^® ^0.5%) and short-acting IV sedation (Propofol^®^), the posterior aspect of the trapezius muscle is exposed through a sagittally oriented skin incision across the shoulder. While the patient is still anesthetized generally thickened fascia, including septae between bundles of the muscle, are excised from the painful area of the muscle according to preoperative skin markings corresponding to the previously identified key tender points. The dissection is continued anteriorly along the leading edge of the trapezius until the SAN was identified. In most of the cases adhesions were identified between the nerve and the underlying fascia.

The patient was then awakened in order to provide feedback during the exploration of areas of greatest focal pain. The skin had been marked pre-operatively to indicate where the key areas of focal tenderness had been identified previously based upon response to local anesthetic. While awake, patients were asked to identify "old" (preoperative) pain and differentiate it from "new" (surgical) pain.

Patients generally signaled incremental improvement during resection of fascia and/or interfascicular septae within the trapezius. Although the SAN neurolysis in some cases was observed to have a direct effect on the patient's headache complaints, the fasciectomy tended to have a greater immediate effect on mobility.

### Data collection

A linear visual analogue scale graded from 0 (not disabling) to 10 (completely disabling) was used to define a 'disability score' for each of five different indices: pain, headache, insomnia, muscle weakness, and neck/shoulder stiffness. Assessments were made for the week preceding surgery, and at follow-up 12-18 months post op.

Hospital charts were reviewed for information pertaining to surgical technique and findings. Outcome data was compiled from questionnaires completed by the patients 12-18 months after the operation.

### Statistical analysis

Student's *t*-test for paired samples was used for the analysis of outcome data. Statistical significance was defined as *p *≤ 0.05.

## Results

### Patients

The study group consisted of 6 males and 24 females presenting to UNMC for treatment for chronic symptoms after whiplash. The average age at the time of surgery was 43 years (range 27-66). The mean and median time from the onset of symptoms until surgery was 41 months (range 7-156) and 24 months, respectively. All of the patients stated that their condition had reached a steady state at the time of the operation.

Fourteen patients reported that their condition had been precipitated by a classic rear-impact motor vehicle crash. The remaining 16 patients reported various other mechanisms of trauma, including falls and sports injuries.

### Preoperative complaints and clinical findings

Only "pain" was identified by all 30 patients as an independent preoperative reason for disability. Each of the remaining four variables (headaches, sleep deprivation, stiffness, and weakness) was a reason for disability in 26 or more patients prior to surgery (Table 1).

**Table 1:** Reported incidence of five separate symptoms, described as disabling by 30 patients prior to surgery.

*Reason for disability*	*Number of patients*
Pain	30

Headache	27

Sleep deprivation	29

Weakness	26

Stiffness	27

Four clinical signs were documented in all cases prior to surgery: (1) asymmetric posture, typically with the shoulder elevated on the side of greatest pain; (2) decreased and painful range of motion in neck and shoulder(s); (3) tenderness to palpation along the horizontal portion of the upper trapezius muscle; and (4) greater than 50% of reduction of pain and increased mobility following infiltration of 2-3 cc of local anesthetic into 1-3 key areas of focal tenderness in the upper trapezius.

Neck/shoulder stiffness, which was observed but not objectively measured in most patients before surgery (Additional file 1), was understood primarily as an expression of pain inhibition.

### Surgical interventions

Key portions of each operation were performed without anesthesia, in order to allow communication between the patient and the surgical team. Thus, the extent of neurolysis and fasciectomy was routinely defined by patients' direct feedback including functional testing, e.g. of mobility (Additional file 2). The procedures were well tolerated by all participants tolerated the procedures well. Recovery was generally rapid, with most patients mobile and ambulatory within the first postoperative days (Additional file 3). There were no major surgical or postoperative complications.

### Histological findings

No pathologic findings were noted in any specimens that, in a majority of patients, were submitted for routine microscopy.

### Long-term results

Eighteen patients (60%) reported improvement in all 5 of the assessed indices (neck pain, headaches, insomnia, weakness and stiffness) and an additional 10 patients (33%) reported improvement in at least one parameter, for a total of 93% of patients reporting a lasting positive outcome one year or more following the surgery. One patient did not report any benefit from the operation, noting that her condition was unchanged. Another patient reported increased stiffness after the operation, but at the same time noted that three other symptom areas had improved (Table 2).

**Table 2:** Changes in symptom-derived disability scores at follow up one-year after surgery.

	Percent change					
	0%	1 < 30%	30% < 50%	50% <100%	100%	*n*
**Pain**	2	0	9	13	6	30

**Headache**	2	1	2	12	10	27

**Insomnia**	10	2	3	10	4	29

**Weakness**	4	2	5	11	4	26

**Stiffness**	1	1	6	12	8	28

Calculations based on patients' assessments (VAS; 0% = no improvement, 100% = complete improvement). Whereas 27 patients reported stiffness as a reason for disability before surgery, the number increased to 28 after the operation. One patient who experienced more stiffness after surgery is represented as "0% improvement".

**Figure 2:**
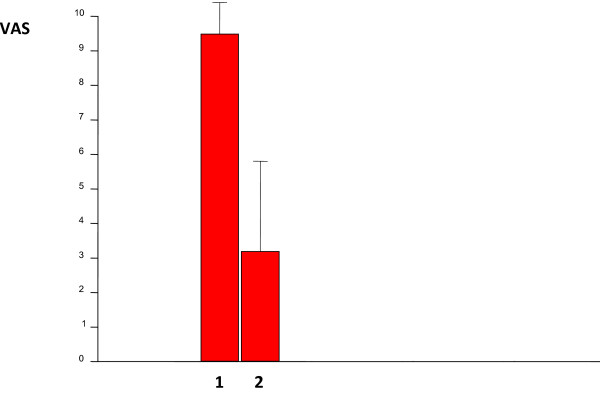
**VAS-scores for global pain before (1) and one year after (2) surgery**. n = 30.

Mean VAS-scores were significantly lower than before surgery for all five variables (Table 3). Specifically, the score for over-all pain decreased from 9.5 ± 0.9 to 3.2 ± 2.6 (Figure 2).

**Table 3:** Symptom-derived disability scores before surgery, and one year after surgery.

*Symptom*	*Before*	*After*	p
**Pain**	9.5 ± 0.9	3.2 ± 2.6	< 0.001

**Headache**	8.2 ± 2.9	2.3 ± 2.8	< 0.001

**Insomnia**	7.5 ± 2.4	3.8 ± 2.8	< 0.001

**Weakness**	7.6 ± 2.6	3.6 ± 2.8	< 0.001

**Stiffness**	7.0 ± 3.2	2.6 ± 2.7	< 0.001

Numerical values represent patients' self-assessments, using a linear Visual Analogue Scale (VAS) graded 0-10 for 0 = "Nothing at all" and 10 = "Completely disabling".

**Figure 3:**
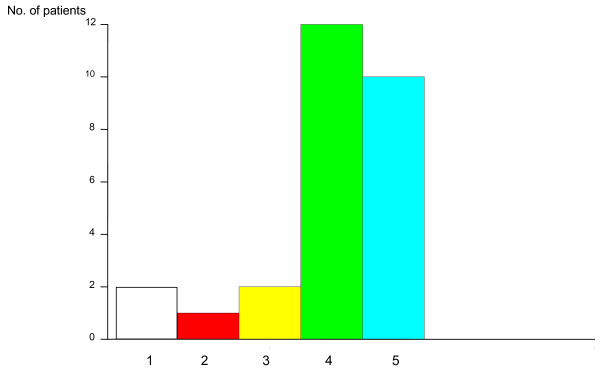
**Impairment caused by headache (VAS) at one-year follow-up relative to pre-surgical symptoms**. 1 = No change; 2 = < 30% reduction; 3 = 30-49% reduction; 4 = 50-99% reduction; 5 = complete resolution. n = 27.

Prior to the operation, 27 patients complained of head pain/headache. After surgery 22 patients stated that their head pain had been reduced by at least 50%, and 10 of these patients stated their headaches had been completely eliminated (Figure 3). Of the 17 patients who continued to experience headaches, all reported that the episodes were less frequent than before surgery, a reduction from 5 ± 2 days/week to 1.4 ± 2 days/week.

Fourteen patients of 29 stated that the severity of their sleep deprivation (insomnia) had decreased by 50% or more as a result of the operation. The average number of hours of sleep per night increased from 4 ± 1 to 6 ± 2 for the entire cohort. Similarly, postoperative perception of disability decreased by at least 50% in 15 of the 26 patients reporting weakness, and in 20 of the 28 patients reporting stiffness, in comparison with pre-operative perceptions.

## Discussion

We describe the long-term outcome after surgical fasciectomy and SAN neurolysis for symptoms associated with chronic whiplash pain. In this series of 30 patients, 29 described a lasting overall improvement that they attributed to the treatment, although in one case with satisfactory pain reduction, the procedure resulted in increased neck stiffness. One patient who did not benefit from the surgery reported no degradation or other worsening of symptoms or disability during the year following the operation.

Our results suggest that some of the most common symptoms found in chronic whiplash (e.g. headaches, stiffness of the neck, and pain in the shoulder/neck region) may be secondary to either primary injury in, or secondary dysfunction of the spinal accessory nerve and/or the trapezius muscle. We conclude, with caution, that the condition represents an indication for surgical treatment in selected cases where more conservative measures have proven ineffectual.

What is less clear is how or why the trapezius muscle and SAN are involved in perpetuating the chronic whiplash syndrome. The traditional portrayal of SAN as one of pure motor function has been challenged by anatomical studies [20,21], and our experience with surgical manipulation of the nerve in alert and unanesthetized patients has confirmed that it indeed is one of mixed sensory and motor function. This finding raises the possibility of SAN injury or entrapment as a cause of neurogenic pain, in addition to and independent of gross loss of motor function [22-24]. Previous reports that surgical neurolysis alone can provide immediate relief of symptoms related to a lesion of SAN further suggest entrapment by scar tissue, rather than nerve damage *per se*, as a reason for some preoperative symptoms [19,25].

Based upon the present data we cannot discern to what extent preoperative symptoms were expressions of dysfunction in the SAN versus the trapezius muscle and/or fascia. It has been reported, however, that patients with chronic whiplash syndrome exhibit higher EMG activity in the upper trapezius muscles than healthy control subjects, as well as a reduced ability to relax the muscle to baseline levels after a dynamic task [26]. Larsson et al. found that chronic neck pain may be associated with disturbed microcirculation in the trapezius [27], and Hagert et al. presented clinical data suggesting chronic trapezius ischemia in a chronic pain syndrome nearly identical to that of our patients [18]. Thus, we cannot exclude that the most beneficial part of the surgery described herein was decompression of a chronic compartment syndrome in parts of the segmented trapezius muscle.

## Limitations

The conclusions that can be drawn from this investigation are limited by the size of the study group, the retrospective, non-randomized study design, and the subjective assessment instrument. It is not possible to draw a firm conclusion as to the relative importance of fasciectomy versus neurolysis, since dissection of the SAN was necessary in all patients to protect the nerve during resection of fascia from the ventral aspect of the trapezius.

## Conclusions

The results described herein offer a potentially new direction in evaluation and surgical treatment of chronic whiplash syndrome. Entrapment of the spinal accessory nerve and/or chronic compartment syndrome of the trapezius muscle may cause chronic debilitating pain after whiplash trauma, without radiological or electrodiagnostic evidence of injury. In such cases, surgical treatment may provide lasting relief. Continued research using randomized and controlled study designs will further advance the understanding and extrapolability of the present findings.

## Abbreviations

SAN: Spinal accessory nerve

## Competing interests

The authors declare no competing interests. No external funding was received for this research.

## Authors' contributions

All coauthors participated in two or more key elements (study design, data collection, analysis of data, manuscript preparation) of this investigation, and read/approved the final manuscript.

## Supplementary Material

Additional file 1 **Pre-operative shoulder function**. Video documentation of shoulder range of motion before surgery. Limited range of motion in right shoulder prior to surgery, in a patient with 10-year history of chronic whiplash from a motor vehicle crash. Click here for file

Additional file 2 **Patient feed-back during surgery**. Video documentation of surgical procedure. Functional progress during neurolysis of spinal accessory nerve and trapezius fasciectomy. The unanesthetized patient cooperates actively and provides guidance to the surgical team. Click here for file

Additional file 3 **Post-operative shoulder function**. Post-operative status. Video documentation of range of motion in right shoulder one day after trapezius fasciectomy and neurolysis of spinal accessory nerve. Click here for file
